# Molecular classification of selective oestrogen receptor modulators on the basis of gene expression profiles of breast cancer cells expressing oestrogen receptor α

**DOI:** 10.1038/sj.bjc.6600477

**Published:** 2002-08-12

**Authors:** A S Levenson, I L Kliakhandler, K M Svoboda, K M Pease, S A Kaiser, J E Ward, III, V C Jordan

**Affiliations:** Robert H Lurie Comprehensive Cancer Center, Northwestern University Medical School, 303 E. Chicago Avenue, Chicago, Illinois, IL 60611, USA; Department of Mathematical Science, Michigan Technology University, Houghton, Michigan, MI 49931-1295, USA

**Keywords:** SERMs, ERα, gene expression profiles, breast cancer cells

## Abstract

The purpose of this study was to classify selective oestrogen receptor modulators based on gene expression profiles produced in breast cancer cells expressing either wtERα or mutant_351_ERα. In total, 54 microarray experiments were carried out by using a commercially available Atlas cDNA Expression Arrays (Clontech), containing 588 cancer-related genes. Nine sets of data were generated for each cell line following 24 h of treatment: expression data were obtained for cells treated with vehicle EtOH (Control); with 10^−9^ or 10^−8^ M oestradiol; with 10^−6^ M 4-hydroxytamoxifen; with 10^−6^ M raloxifene; with 10^−6^ M idoxifene, with 10^−6^ M EM 652, with 10^−6^ M GW 7604; with 5×10^−5^ M resveratrol and with 10^−6^ M ICI 182,780. We developed a new algorithm ‘Expression Signatures’ to classify compounds on the basis of differential gene expression profiles. We created dendrograms for each cell line, in which branches represent relationships between compounds. Additionally, clustering analysis was performed using different subsets of genes to assess the robustness of the analysis. In general, only small differences between gene expression profiles treated with compounds were observed with correlation coefficients ranged from 0.83 to 0.98. This observation may be explained by the use of the same cell context for treatments with compounds that essentially belong to the same class of drugs with oestrogen receptors related mechanisms. The most surprising observation was that ICI 182,780 clustered together with oestrodiol and raloxifene for cells expressing wtERα and clustered together with EM 652 for cells expressing mutant_351_ERα. These data provide a rationale for a more precise and elaborate study in which custom made oligonucleotide arrays can be used with comprehensive sets of genes known to have consensus and putative oestrogen response elements in their promoter regions.

*British Journal of Cancer* (2002) **87**, 449–456. doi:10.1038/sj.bjc.6600477 www.bjcancer.com

© 2002 Cancer Research UK

## 

Selective oestrogen receptor (ER) modulators (SERMs) are a new class of drugs, which have a profound impact in breast cancer treatment and prevention (Levenson and Jordan, 1999). Tamoxifen is one of the most effective SERM for the treatment of ER positive breast cancers; however, despite the initial favourable response to tamoxifen therapy, eventually tumours become refractory to treatment resulting in disease recurrence (Tonetti and Jordan, 1995). Often these tumours will subsequently respond to alternative hormonal therapy.

It is well known that there are mechanistic differences among SERMs because following binding to ER each ligand induces a distinct ER-ligand conformation, that is recognised distinctly by the transcription machinery (Wijayaratne *et al*, 1999). It is, therefore, logical to assume that individual SERMs induce different gene expression profiles. Identification of additional SERMs, on the basis of their differential gene expression profiles, could be useful in predicting outcome of the second-line therapy.

Differential pharmacology of SERMs is best represented in different tissues. Table 1

**Table 1:**
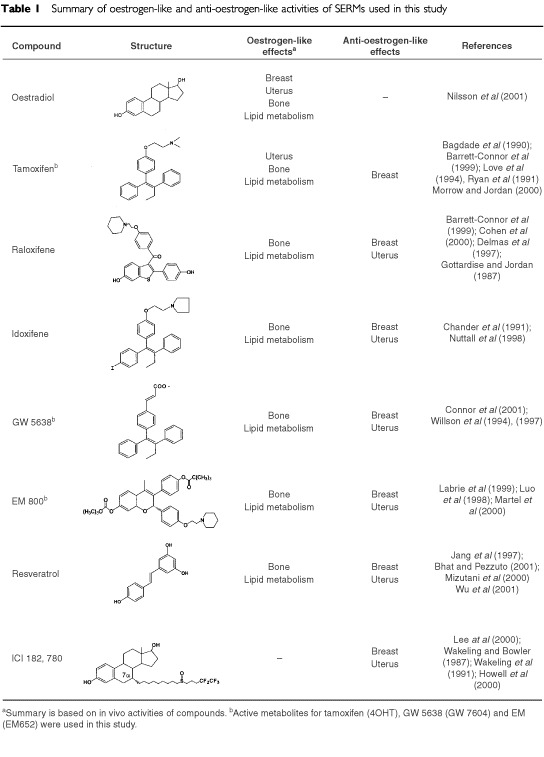
Summary of oestrogen-like and anti-oestrogen-like activities of SERMs used in this study

summarises the *in vivo* pharmacological and/or therapeutic activities of compounds used in this study. The mechanisms of oestrogen action by binding two specific intracellular ERs (α and β) reviewed in (Nilsson *et al*, 2001). The anti-oestrogen tamoxifen has oestrogenic activities in the bone, uterus and the cardiovascular system (Bagdade *et al*, 1990; Ryan *et al*, 1991; Love *et al*, 1994; Barrett-Connor *et al*, 1999) and is an anti-oestrogenic in the breast (Jordan, 2000; Morrow and Jordan, 2000). Raloxifene (Ral), on the other hand, has less oestrogenic effects on uterus, but the same beneficial effects on bone and lipid metabolism (Delmas *et al*, 1997; Barrett-Connor *et al*, 1999; Cohen *et al*, 2000) while it is an antagonist of oestrogen action in the breast (Gottardis and Jordan, 1987; Cummings *et al*, 1999). Other SERMs like idoxifene (Idox) (Chander *et al*, 1991; Nuttall *et al*, 1998), GW 5638 (Willson *et al*, 1994, 1997; Connor *et al*, 2001) and EM 800 (Luo *et al*, 1998; Labrie *et al*, 1999; Martel *et al*, 2000) are different from tamoxifen and based on their activity in the rodent uterus are more closely related to raloxifene. Resveratrol (Res) does not formally belong to the SERM family, because it is a phytoestrogen found in the plants (Jang *et al*, 1997), however Res has similar *in vivo* characteristics to the SERMs (Mizutani *et al*, 2000; Bhat and Pezzuto, 2001; Wu *et al*, 2001). ICI 182,780 (ICI) is a pure anti-oestrogen with anti-oestrogenic activities in almost all tissues, with no changes in serum lipids and on bone density (Wakeling and Bowler, 1987; Wakeling *et al*, 1991; Lee *et al*, 2000). ICI 182,780 is currently in Phase III clinical trials for the treatment of advanced breast carcinoma (Howell *et al*, 2000).

Although it is possible to determine quantitatively the expression of thousands of genes in a single sample, there are significant technical issues to address for a comparison of gene expression profiles induced by a SERM-ER complex in different tissues. Cellular heterogeneity of different tissues is one of the problems. An approach to this issue would be to use a system *in vitro* to characterise different compounds and their differential pharmacology. We have developed a model system *in vitro*, in which some SERMs demonstrate oestrogen-like activities while others remain anti-oestrogenic (Levenson *et al*, 1997, 1998a,b,c, 2001; Levenson and Jordan, 1998).

The goal of this study was to classify SERMs on the basis of gene expression profiles of breast cancer cells expressing either wtERα (D351) or mutant_351_ERα (D351Y) after exposure to SERMs for 24 h. To our knowledge this is the first attempt to classify SERMs using cDNA microarrays. We chose a well characterised model system *in vitro* in which cellular machinery is adapted for ER-nonmediated transcription of genes (MDA-MB-231 cells), and oestrogen-responsiveness is restored by introduction of the ER gene into cells. The D351Y mutation in ER, changes SERM-induced transcriptional activities of endogenous gene expression (Levenson *et al*, 1997, 1998b,c, 2001; Levenson and Jordan, 1998; Macgregor Schafer *et al*, 1999) in these cells.

Different analytical tools have been proposed for analysing microarray data (Eisen *et al*, 1998; Golub *et al*, 1999; Kalocsai and Shams, 2001; Tamayo *et al*, 1999; Toronen *et al*, 1999; Bittner *et al*, 2000). Our goal was to identify the gene expression profiles of cells treated with the drug, compare the profiles from all samples (cells treated with different drugs) and then cluster similar from different profiles. We describe a new algorithm ‘Expression Signatures’ for the classification of compounds based on differential gene expression profiles of cells treated with these compounds.

## MATERIALS AND METHODS

### Tissue culture

The cells stably expressing wtERα and mutant_351_ERα used in this study were constructed from the ER-negative MDA-MB-231 human breast cancer cells as described previously (Jiang and Jordan, 1992; Catherino *et al*, 1995). The level of ERα in both cell lines is comparable to the level of ERα in MCF-7 cells. Cells were maintained in phenol red-free MEM with 5% charcoal-dextran treated calf serum, supplemented with 100 μg ml^−1^ streptomycin, 100 units ml^−1^ penicillin, 2 mM L-glutamine, 6 ng ml^−1^ bovine insulin, 100 mM nonessential amino acids, and 500 μg ml^−1^ G418. All of the tissue culture solutions were from Life Technologies, Inc. (Gaithersburg, MD, USA).

### Hormones

17-β Oestradiol (E2) and Res were purchased from Sigma Chemical Co. (St. Louis, MO, USA). 4-OHT, EM-652 and ICI 182,780 were gifts from Dr Alan Wakeling (ICI Pharmaceuticals Macclesfield, UK). Raloxifene was a generous gift from Eli Lilly Research Laboratories (Indianapolis, IN, USA). GW 7604 was a generous gift from Dr Timothy Willson (Glaxo Wellcome Inc., Durham, NC, USA). Idoxifene was a gift from Smith Kline Beecham (Philadelphia, PA, USA). All compounds used in the experiments were dissolved in 100% ethanol and added to the medium in 1 : 1000 dilutions for a final ethanol concentration no higher than 0.2%.

### cDNA array-based expression profiling

We used Atlas™ Human cDNA Expression Arrays (Clontech, Palo Alto, CA, USA), containing cDNA fragments representing 588 known cancer-related genes and nine housekeeping genes. A complete list of the 588 genes of the Atlas™ Human Expression Array used can be accessed through the Internet at http://www.clontech.com.

Total RNA was isolated using TRIZOL reagent (Gibco–BRL) as described previously (Levenson *et al*, 1997). The preparation and hybridisation of ^32^P-labelled cDNA from 5 μg of total RNA were performed as described in the manufacturer's protocol. The experiments for each condition were conducted three times with at least two sets of RNAs and two different membranes. The hybridised membranes were exposed for 72–96 h at −80°C using BioMax TranScreen HE Intensifying Screen (Kodak, Rochester, NY, USA). Signal intensities at each cDNA spot were detected by phosphorimage analysis using a Molecular Dynamic Storm phosphoimager (Molecular Dynamics, Sunnyvale, CA, USA). Initial analysis was performed using AtlasImage™ 1.5 software (Clontech). Following background correction, each gene on the membrane was normalised to a reference GAPDH then the signal intensity was calculated for each spot on the array and reported in an Excel database. For further analysis of the data (see below) ‘all to one’ normalisation was applied when all samples (treated with compounds) were normalised to one sample (untreated control).

### ‘Expression signatures’ analysis

A new method for classification of different compounds on the basis of gene expression profiles entitled ‘expression signatures’ was developed by our group. The method involved the following steps. Genes were reordered in the ascending order of their expression levels in the untreated samples. We denoted the expression of a gene with number ‘i’ in reordered set as E_i,_ where sub-index ‘i’ corresponds to the gene number, and E_i_ means an expression level of that gene. Therefore, E_j_ >E_i_ for j >i. Further, the data in gene arrays obtained under the influence of compound R were arranged in the same order, and were denoted as E_i_^(R)^. We propose the following formula to compute rescaled gene expressions P_i_:


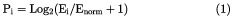


E_norm_ is normalizing coefficient. In the present work, we adopted the following expression for E_norm_: E_norm_=E_ref_/E_min_^1/2^. We used GAPDH as a reference (E_ref_), and E_min_ is the minimal level of intensity identified in the experiments. If sets of E_i_ correspond to the untreated sample, we get sets of P_i_^(0)^ of scaled gene profiles; for samples treated with compound R, we obtain P_i_^(R)^. The formula (1) has two important properties: (a) original expressions E_i_=0 are transformed into rescaled P_i_=0, and (b) for high E_i_ the formula is asymptotically logarithmic with base 2. Next, we plot P_i_^(R)^ for each compound *versus* P_i_^(0)^ of untreated sample. This is called the ‘expression signature’ of the compound. Similarities or differences between gene expression profiles produced by compounds R and Q can be calculated using Euclidian distance D [R,Q]:


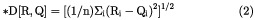


where n is total number of genes. Interpretation of the distances and signatures allows grouping of the compounds on the basis of gene expression profiles produced.

The intrinsic gene list that formed the basis for the classification was selected for each cell line to include those which showed good reproducible expression data from each of the three experiments performed for each compound and those which had intensity higher than 4000. This subset of genes for cells expressing wtERα was represented by 87 genes and for those expressing mutant_351_ ERα by 117 genes. (Complete data sets for expression profiles are available at http://www.math.mtu.edu/∼igor/Gene_index.html.)

### Cluster analysis

We extracted normalised expression data for cells expressing wtERα from an Excel data base generated by AtlasImage™ 1.5 software (Clontech). The same criteria of reproducibility and accuracy were applied to select subsets of genes from the 588 cDNAs on the array. Clustering was performed using the subset of up-regulated genes after treatment with compounds compared to the control untreated cells.

We applied a hierarchical clustering algorithm described by Eisen *et al* (1998) and available as ‘Cluster and TreeView’ manual at http://rana.stanford.edu/software. The results of this process were two dendrograms, one for the compounds and one for the genes. The dendrogram for the compounds is of interest for the current study.

## RESULTS

We identified gene expression profiles in MDA-MB-231 human breast cancer cells transfected with either wtERα or mutant_351_ERα following treatment with E2, SERMs (4OHT, Ral, Idox, GW, EM, Res) and pure anti-oestrogen ICI. In total, 54 microarray experiments were carried out. Nine sets of data were generated for each cell line following 24 h of treatment: gene expression profile for cells treated with vehicle EtOH (Control); with 10^−9^ or 10^−8^ M E2; with 10^−6^ M 4OHT; with 10^−6^ M Ral; with 10^−6^ M Idox, with 10^−6^ M EM, with 10^−6^ M GW; with 5×10^−5^ M Res and with 10^−6^ M ICI. The concentrations of compounds for each cell line used in this study have been determined previously (Levenson *et al*, 1997; 1998a,b,c, 2001) and the maximally effective dose for each compound was chosen for the array experiments.

**Figure 1:**
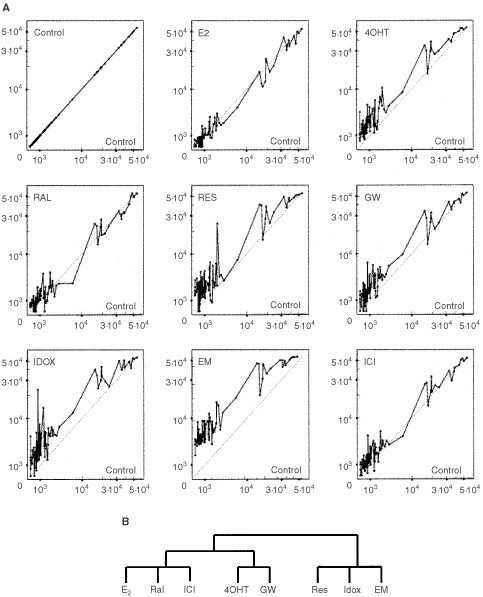
Expression signatures of cells treated with different compounds (E2, SERMs and ICI) *versus* the untreated control cells are shown. Eighty-seven selected genes were used to create ‘Signatures’ for cells expressing wtERα (**A**) and dendrogram representing similarities in the expression patterns of cells treated with different compounds were created from ‘expression signatures’ on the basis of data from distance metric and correlation coefficients (**B**). The branching patterns in the resulting dendrogram organised the compounds into three main groups: E2 : Ral : ICI; 4OHT : GW; and Res : Idox: EM. Normalised values for selected subset of genes were used for all manipulations. The values of normalised adjusted intensities representing levels of expressions of the vehicle-treated control (X-axis) and the compound-treated (Y-axis) cells are shown for **A**.

**Figure 2:**
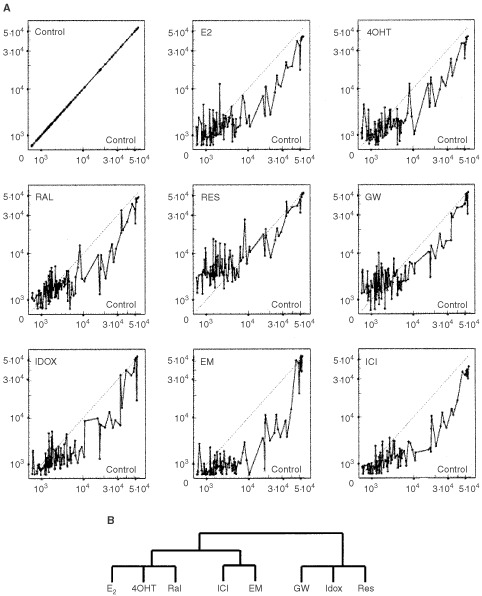
Expression signatures of cells treated with different compounds (E2, SERMs and ICI) *versus* the untreated control cells are shown. One hundred and 17 selected genes were used to create ‘Signatures’ for cells expressing mutant_351_ERα (D351Y) (**A**) and dendrogram representing similarities in the expression patterns of cells treated with different compounds were created from ‘expression signatures’ on the basis of data from distance metric and correlation coefficients (**B**). The branching patterns in the resulting dendrogram organised the compounds into three main groups: E2 : 4OHT : Ral; ICI : EM; and GW : Idox: Res. Normalised values for selected subset of genes were used for all manipulations. The values of normalised adjusted intensities representing levels of expressions of the vehicle-treated control (X-axis) and the compound-treated (Y-axis) cells are shown for **A**.

We developed a new algorithm ‘expression signatures’ (see Materials and Methods) to classify compounds based on differential gene expression modulation. In contrast to the hierarchical cluster analysis this method allows the use of raw normalised data sets without any major modification of the data. We first considered the whole set of 588 genes represented on the array to compare the ‘signatures’ of compound-treated cells. However, based on our findings of unacceptable experimental variations and artefacts produced by the microarray technology itself, we searched for genes that were not influenced by the experimental variations (see Materials and Methods). Figure 1A

shows ‘expression signatures’ of cells expressing wtERα treated with different compounds for 24 h. Interpretation of the signatures on basis of calculated ‘distances’ between the compounds showed similarities and differences between expression profiles of studied compounds. The resulting dendrogram for wtERα (Figure 1B) produced with a selected subset of genes (87 genes) (see Materials and Methods), resembled closely the dendrogram with all 588 genes (data not shown). Surprisingly, E2, Ral and ICI grouped together, the next closest is the group containing 4OHT and GW, whereas Res, Idox and EM were on the most distant dendrogram branches. This means that EM had lower correlation to the Control untreated cells, whereas E2 had the highest correlation to the untreated cells. When 117 selected groups of genes were used for ‘expression signatures’ of cells expressing mutant_351_ERα, the resulting dendrogram (Figure 2A,B

) showed slightly different branching patterns. This time the most distant branches from the E2, 4OHT and Ral group was the GW, Idox and Res group.

**Figure 3:**
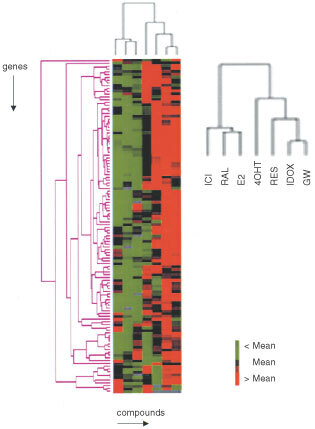
Gene expression patterns of cells expressing wtER related to treatment with compounds (E2, SERMs and ICI). Two-dimensional hierarchical clustering was applied to up-regulated subset of expression data from a total of 588 cDNAs measured across seven different treatments. A cluster dendrogram representing the hierarchical relationships between gene expression profiles of cells (vertically) and compounds (horizontally) was then generated. Colour-coded gene expression values for genes are shown. The colour reflects the mean-adjusted expression level of the gene: black is the mean, red is greater than the mean and green is less than the mean.

The hierarchical clustering (Eisen *et al*, 1998) is often used as a multivariate technique to find groups of genes with similar expression profiles across a number of experiments and to group the experimental samples according to the similarities in their overall patterns of gene expression. This method was successfully applied for tumour, tissue or cancer cell line classification purposes (Ben-Dor *et al*, 2000; Perou *et al*, 2000; Ross *et al*, 2000; Gruvberger *et al*, 2001; Sorlie *et al*, 2001; Welsh *et al*, 2001). To examine the robustness of the observed clustering patterns by ‘Expression signatures’, hierarchical cluster analysis (see Materials and Methods) was performed by using subsets of up-regulated genes for each of seven experimental conditions for cells expressing wtERα (Figure 3

). Once again, E2, Ral and ICI clustered together while cells treated with 4OHT, Res, Idox and GW formed another big branch of the dendrogram.

Therefore, this data suggests that the classification of SERMs is relatively robust with E2, Ral and ICI staying together in the same cluster when using different sets of selected genes for analysis.

## DISCUSSION

The biological effects of oestrogen and SERMs in breast cancer are mediated by the ER, a transcriptional regulator that controls the pattern of gene expression. The crystal structures of the ligand binding domain (LBD) of wtERα with agonists (E2 and DES) and antagonists (Ral and 4OHT) (Brzozowski *et al*, 1997; Shiau *et al*, 1998) confirmed the central role of the ligand in modulating ER conformation. It is now clear that each ligand upon binding, induces unique structural alterations within the ER, changing its conformation and its transcriptional activity. This means that subsequent gene expression results from the ER-SERM complex activating different, but complementary signal transduction pathways.

Because the actual mechanism that leads to differences of final phenotype depend not on single genes but on a global expression pattern, we used cDNA Atlas Arrays to evaluate gene expression profiles after activation of ERα by different ligands. It has been previously shown that it is possible to group SERMs based on differences and similarities in the pattern of gene expression changes that they produced (Zajchowski *et al*, 2000). The authors evaluated 24 gene per cell combinations comprising 10 different known oestrogen-responsive genes in eight cell lines representing four cell types. The aim of this study was to examine SERMs on the basis of gene expression profiles using cDNA microarrays in breast cancer cells known to elicit various oestrogenic responses to different compounds.

It is very important to emphasise that in general the differences between gene expression profiles treated with different compounds were very small with correlation coefficients ranging from 0.83 to 0.98. Our results reflect several limitations of this approach: (i) we treat the same cell line (not a different cell line from different origin) with compounds, so the differences are already very limited; (ii) all these compounds (except ICI) belong to one class of drugs (SERMs) that function through the ER, i.e. have similar mechanism of action; and (iii) very small amount of all human genes are represented on the arrays used. By analogy, when 20 of the tumours were sampled before and after chemotherapy (Perou *et al*, 2000) clustering analysis indicated a high degree of similarity within samples derived from the same patient compared with those from different patients. As a consequence, for tumour classification, Sorlie *et al* (2001) have used the list of genes to include those with significantly greater variation in expression between different tumours than between paired samples from the same tumour.

Our previous classification of SERMs in these two cell lines was based on one single marker of E2-responsiveness in these breast cancer cells: E2 and 4OHT produced oestrogenic effects on the activation of TGFα mRNA in cell expressing wtERα whereas Ral and ICI were anti-oestrogens (Levenson *et al*, 1998b). With the mutant receptor, Ral became oestrogenic as well (Levenson *et al*, 1997; Levenson and Jordan, 1998). These observations were extremely important for understanding SERM action once X-ray crystallography of the LBD of ERα with Ral and 4OHT was determined (Brzozowski *et al*, 1997; Shiau *et al*, 1998). Resveratrol had the same effects on growth and expression of selected target genes as E2 in both cell lines (Levenson, unpublished data). Both GW and EM, on the other hand, showed similar effect as Ral with D351 but were weakly oestrogenic with mutant D351Y ER (Macgregor Schafer *et al*, 1999; Bentrem *et al*, 2001; Levenson *et al*, 2001). It is hard to predict what impact on classification of SERMs would TGFα have, being just one gene out of 588 cDNA spotted on the arrays, but because the expression of TGFα in all membranes was at background levels, it was not considered at all. This is concern with the technology. Despite this major limitation we proceeded with a global analysis of results.

The most unexpected result was the fact that E2 grouped with Ral and ICI in cells expressing wtERα. With presumed mechanism of action for pure anti-oestrogen ICI, we expected ICI to appear at the most distant branches of dendrogram from E2. The mode of action of ICI recently summarised by Howell *et al* (2000) is that ICI binds to ER with affinity similar to that of E2, triggers rapid degradation of ER, therefore there is a reduced rate of dimerisation and nuclear localisation of ICI-ER complex with reduced binding of the complex to oestrogen response elements (EREs). There is no precise molecular description of those events, but obviously reduced rate of ER-ICI complex and different conformational changes in the dimmer influence various protein–protein interactions in the transcriptional complex with subsequent transcription of non-ER-mediated genes as well. Although the mode of action of E2, Ral and ICI is different in terms of direct activation of E2-responsive target genes with EREs (Howell *et al*, 2000), it might be more similar in terms of transcription of other genes. It is known that differences in ERE sequence also impact ER binding affinity and transcriptional activity: there is enhanced transcription with ‘direct binding’ to the consensus ERE and ‘tethering’ mechanism where ER interacts with another DNA-bound transcription factor (i.e. Sp1, Ap-1) to initiate responsiveness of other target genes (Klinge, 2001; Nilsson *et al*, 2001). Overall, if we consider that (1) the level of ER protein goes down with E2, Ral and ICI treatments in these cells (Levenson *et al*, 1998c; Liu *et al*, 2001) and (2) the Atlas Arrays we used were spotted with PCR-amplified cDNAs for 588 cancer-related genes with a very few E2-responsive genes with putative EREs in their promoter regions, it is fair to say that we are only detecting changes in non-ER-mediated genes. Among E2-responsive genes were TGFα, VEGF, BRCA1 on the array. We were surprised to find that ligand-enhanced activation of these genes was only background and we could not obtain significant variations in expression among SERMs-treated cells. Clearly, future ventures to discover signal transduction pathways must employ targeted array technology. Custom build gene arrays specifically designed to address the cell cycle, apoptosis and angiogenesis and replete with oestrogen-responsive genes may be the only way to address mechanistic questions.

Finally, we were interested to observe that SERMs up-regulated genes compared to control untreated cells in cells expressing D351 (Figure 1A) whereas in contrast, SERMs down-regulated genes in cells expressing mutant D351Y ER (Figure 2A). The fact that the ER is known to cause a repressive action with oestrogen in transfectants (Levenson and Jordan, 1994) that is entirely opposite from the actions of oestrogen in nature, suggests that SERMs are doing the same with the D351Y ER where responses are more oestrogen-like. Less complex cell systems should be addressed in the future.
